# Microglia Inhibition Delays Retinal Degeneration Due to MerTK Phagocytosis Receptor Deficiency

**DOI:** 10.3389/fimmu.2020.01463

**Published:** 2020-07-16

**Authors:** Deborah S. Lew, Francesca Mazzoni, Silvia C. Finnemann

**Affiliations:** Department of Biological Sciences, Center for Cancer, Genetic Diseases and Gene Regulation, Fordham University, Bronx, NY, United States

**Keywords:** retinitis pigmentosa, MerTK, phagocytosis, microglia, retinal degeneration

## Abstract

Retinitis Pigmentosa (RP) is a group of inherited retinal diseases characterized by progressive loss of rod followed by cone photoreceptors. An especially early onset form of RP with blindness in teenage years is caused by mutations in *mertk*, the gene encoding the clearance phagocytosis receptor Mer tyrosine kinase (MerTK). The cause for blindness in mutant MerTK-associated RP (mutMerTK-RP) is the failure of retinal pigment epithelial cells in diurnal phagocytosis of spent photoreceptor outer segment debris. However, the early onset and very fast progression of degeneration in mutMerTK-RP remains unexplained. Here, we explored the role of microglia in the Royal College of Surgeons (RCS) rat model of mutMerTK-RP. We found elevated levels of inflammatory cytokines and CD68 microglia activation marker, and more ionized calcium-binding adapter molecule 1 (Iba-1) positive microglia in RCS retina when compared to wild-type retina as early as postnatal day 14 (P14). Strikingly, renewal of photoreceptor outer segments in P14 wild-type rat retina is still immature with low levels of RPE phagocytosis implying that at this early age lack of this process in RCS rats is unlikely to distress photoreceptors. Although the total number of Iba-1 positive retinal microglia remains constant from P14 to P30, we observed increasing numbers of microglia in the outer retina from P20 implying migration to the outer retina before onset of photoreceptor cell death at ~P25. Iba-1 and CD68 levels also increase in the retina during this time period suggesting microglia activation. To determine whether microglia affect the degenerative process, we suppressed retinal microglia *in vivo* using tamoxifen or a combination of tamoxifen and liposomal clodronate. Treatments partly prevented elevation of Iba-1 and CD68 and relocalization of microglia. Moreover, treatments led to partial but significant retention of photoreceptor viability and photoreceptor function. We conclude that loss of the phagocytosis receptor MerTK causes microglia activation and relocalization in the retina before lack of RPE phagocytosis causes overt retinal degeneration, and that microglia activities accelerate loss of photoreceptors in mutMerTK-RP. These results suggest that therapies targeting microglia may delay onset and slow the progression of this blinding disease.

## Introduction

Retinitis Pigmentosa (RP) is a heterogeneous group of inherited retinal degenerations that show rod photoreceptor defects and loss before secondary loss of cone photoreceptors resulting in blindness (1). To date, mutations in over a hundred genes mostly expressed in rod photoreceptors or the adjacent retinal pigment epithelium (RPE) are known to lead to RP (2, 3). Although RP may progress at different rates even among patients with mutations in the same gene, usually rod dysfunction leads to night blindness in late teenage age followed by rod death by apoptosis and loss of peripheral vision (tunnel vision). Eventually, secondary “bystander” death of cone photoreceptors will lead to blindness usually in middle age (1). Mutations of the *mertk* gene encoding the Mer receptor tyrosine kinase (MerTK) cause an exceptionally severe form of RP in human patients with childhood onset and blindness in teenage years (4–10). No therapy is available to date for mutant MerTK-associated RP (mutMerTK-RP) that will prevent or even delay progression to blindness.

Disease manifestation in mutMerTK-RP has been elucidated exploring animal models that mimic well the human disease. The Royal College of Surgeons (RCS) rat strain was recognized as model retinal degeneration in the 1960's and has since been studied extensively (11). The RCS rat genome carries a deletion in the coding sequence of the *mertk* gene resulting in an aberrant transcript encoding only 20 of 999 amino acids (12, 13). No MerTK protein is expressed and thus RCS rats are a natural null strain for MerTK. Acute re-expression of MerTK significantly but not completely decreases the severity of RCS rat retinal degeneration (14–16). Mice engineered to lack *mertk* gene activity (*mertk*^−/−^ mice) fully phenocopy the RCS rat further confirming that loss of functional MerTK causes the RCS rat RP phenotype (17).

Mechanistically, MerTK functions as engulfment receptor in non-inflammatory apoptotic cell clearance phagocytosis, a specialized form of phagocytosis also known as efferocytosis. As essential aspect of the life-long and continuous process of photoreceptor outer segment renewal, clearance phagocytosis by the RPE removes spent photoreceptor outer segment tips from the outer retina in a strict diurnal rhythm (18, 19). MerTK-deficient RPE cells fail to engulf spent outer segment tips (20, 21) causing outer segment debris to build up in the outer retina where it is thought to distress photoreceptors such that they die by apoptotic cell death (22). Outer segment renewal including daily clearance phagocytosis in rodents commences after postnatal formation of photoreceptor outer segments and has been assumed to be associated with retinal maturation around eye opening, which in our RCS rats occurs at postnatal day 15 (P15) plus or minus one day. No significant debris buildup is observed before P22, yet rod photoreceptor apoptosis is substantial only shortly thereafter, at ~P25 (22, 23). Retinal function as measured by electroretinography shows normal photoreceptor response at eye opening, and modestly and severely reduced photoreceptor responses at P22 and P34, respectively (11). Altogether, it has long been assumed that photoreceptor degeneration in mutMerTK-RP occurs only after eye opening, subsequent to postnatal retinal maturation and as consequence of defective RPE phagocytosis. Here, we investigate whether inflammatory mechanisms contribute to its remarkably early onset and rapid speed of progression.

Recent studies investigating the role of microglia in several forms of retinal degeneration suggest a dual role for these highly mobile cells. Upon acute damage, as following retinal detachment, microglia may prevent photoreceptor death acting in a protective role (24). Conversely, in chronic damage, such as seen in hereditary retinal dystrophies in mouse models, microglia may contribute to retinal cell death and accelerate retinal degeneration (25–28). In late stage human and rodent RP increased levels of activated microglia have been reported (29, 30). Likewise, in both mouse and rat mutMerTK-RP, microglia activation have been reported at P35 and P50, the late stage of the disease (31, 32). Strikingly, mice constitutively lacking the pro-inflammatory cytokine CCL3 in addition to MerTK (*mertk*^−/−^ *ccl3*^−/−^ double knockout mice), exhibit decreased retinal microglia activation and retain more photoreceptor cells than *mertk*^−/−^ mice by P56 but only this advanced stage of the disease was tested (33). Moreover, RCS rats show activated microglia in the photoreceptor layer of the retina at P21, an early stage of disease (34). Altogether, these intriguing data suggest that microglia may play a role in mutMerTK-RP. Here, we asked if microglia activation has a primary role in the early onset of retinal degeneration due to MerTK deficiency. We found that molecular markers indicating microglia activation are already elevated at P14, an age just before eye opening when RPE phagocytosis of photoreceptor outer segment fragments has not yet reached its mature level, and that microglia relocalize to the photoreceptor layer of the retina starting at P20. We then used three different experimental paradigms to suppress early microglia activation in RCS rats. Assessment of photoreceptor retention and function showed that inactivating microglia at an early age prolonged photoreceptor survival and retinal function.

## Materials and Methods

### Reagents

All reagents were from Millipore-Sigma (St. Louis, MO) or Thermofisher (Carlsbad, CA) unless indicated.

### Animals and Tissue Processing

Animals were housed in a 12-h light/12-h dark light cycle with food and water *ad libitum*. Animals of both sexes were used. Wild-type (WT) and *mertk*^−/−^ mice in the same 129T2/SvEmsJ genetic background (23), pink-eyed dystrophic RCS rats (rdy/rdy-p) and Sprague Dawley (SD) WT rats were raised to yield litters at defined age for experiments. For tissue harvest, animals were euthanized by CO_2_ asphyxiation before immediate eye enucleation and processing. Unless indicated otherwise, all tissue harvest was done at 3–4 h after light onset to avoid variability due to circadian effects. Eyeballs were chilled, dissected and tissue fractions flash-frozen for immunoblotting. For tissue sectioning, cornea and lens were dissected before fixation of tissue in 4% paraformaldehyde in PBS for 30 min followed by sequential dehydration and embedding in paraffin. To generate whole mount preparations of posterior eyecups, cornea, lens and retina were dissected from eyes harvested from WT rats sacrificed 1 h after light onset. Eyecups were fixed in 4% paraformaldehyde in 0.1 M phosphate buffer followed by radial cuts to flatten the eyecup in preparation for immunofluorescence labeling.

### Drug Treatments

To start localized drug treatment before onset of microglia activation we administered tamoxifen (tmx) as eye drops starting at P10. Schlecht et al. previously demonstrated that tmx eye drops applied to closed eyelids of mice from P8 through P12 are sufficient to yield efficient Cre induction in the posterior retina implying that tmx reaches the posterior eye if applied to closed eyelids (35). Here, RCS rats received eye drops of 10 μl of 5 mg/ml tmx in corn oil into both eyes 3 times a day. To administer tmx systemically, rats were fed tmx-supplemented chow (500 mg/kg, Envigo, South Easton, MA, #130858) *ad libitum* starting at weaning (P19). Liposomal clodronate (LC, Liposoma, Amsterdam, The Netherlands) was administered at 10 μl LC/g body weight by intraperitoneal injections every 7 days starting at P13 and at 4 μl /eye by intravitreal injection once the day after eye opening (at P16 or P17). For combined tmx and LC administration, rats received tmx eye drops and the LC treatment as described above. Control siblings were manipulated identically but received corn oil-only eye drops and PBS injections. For all treatments, ERGs were recorded at P33 followed by continued treatment until sacrifice and tissue harvest at P40.

### Electroretinogram (ERG) Recordings

The entire procedure was carried out under dim red light. RCS rats were dark-adapted overnight before intraperitoneal injection of 100 mg/kg ketamine and 10 mg/kg xylazine to induce anesthesia. Scotopic responses were recorded exactly as described previously using a UTAS-E2000 visual electrodiagnostic system (LKC Technologies, Gaithersburg, MD) (23). Stimuli were presented in order of increasing intensity as a series of white flashes of 1.5 cd-s/m2 attenuated with neutral density filters. For each flash intensity, three to six recordings were averaged. For all recordings, a-wave amplitudes were measured from the baseline to the trough of the a-wave, and b-wave amplitudes were measured from the trough of the a-wave to the peak of the b-wave.

### RNA Extraction and RT-PCR

Two dissected neural retinas from a single animal were pooled and processed following the manufacturer's direction using the Qiagen RNeasy Plus Mini kit (Qiagen, Waltham, MA). Purity and concentration of each sample were analyzed by spectrophotometry, and 5 ng/μl RNA stocks were stored at −20°C. RT-PCRs on 10 ng RNA were performed using the Qiagen One-Step RT-PCR kit. Primer sequences are listed in Table 1. Quantification of bands following product electrophoresis was performed using ImageJ.

**Table 1:** RT-PCR primers used.

**Gene rats**	**Primers Forward (F) and Reverse (R)**
**CCL5** (Chemokine C-C motif Ligand 5)	F: 5′-CATCTTCCACAGTCTCTGCTTC R: 5′-GAGCAAGCAATGACAGGAAAG
**GAPDH** (Glyceraldehyde 3-phosphate dehydrogenase)	F: 5′-CTTCTCTTGTGACAAAGTGG R: 5′-GTAGACTCCACGACATACTC
**Gene mice**	**Primers Forward (F) and Reverse (R)**
**CCL2** (Chemokine C-C motif Ligand 2)	F: 5′-AACTCTCACTGAAGCCAGCTC R: 5′-TTAAGGCATCACAGTCCGAGTC
**CCL3** (Chemokine C-C motif Ligand 3)	F: 5′-CAGACACCAGAAGGATACAAGC R: 5′-GGCAGCAAACAGCTTATAGG
**CCL4** (Chemokine C-C motif Ligand 4)	F: 5′-ATGAAGCTCTGCGTGTCTGC R: 5′-CAGAGAAACAGCAATGGTGGAC
**CCL5** (Chemokine C-C motif Ligand 5)	F: 5′-TGCCCTCACCATCATCCTCAC R: 5′-AGGACTAGAGCAAGCAATGACAGG
**Rplp0** (Ribosomal Protein Lateral Stalk Subunit P0)	F: 5′- AGAAACTGCTGCCTCACATC R: 5′-CCCACCTTGTCTCCAGTCTTTATC

### Immunofluorescence Staining and Tissue Analysis

Posterior eyecup whole mount preparations were stained with rhodopsin antibody B6-30 and AlexaFluor488-conjugated secondary antibody to label phagosomes containing photoreceptor outer segment particles, and counterstained with AlexaFluor594-labeled phalloidin and DAPI nuclear dye (36). Phagosome counts were obtained using fixed value of threshold settings in ImageJ including only rhodopsin positive particles with 0.5 μm diameter and above.

Seven micrometer thick microtome sections cut within 200 μm from the optic nerve were deparaffinized. Epitope unmasking was performed by boiling for 10 min in 10 mM citric acid, 0.05% Tween-20, pH 6. Sections were then blocked with PBS, 1% BSA, 0.01% Triton-X100 and incubated sequentially with primary and appropriate AlexaFluor-conjugated secondary antibodies. Primary antibodies used were to ionized calcium-binding adapter molecule 1 (Iba-1) (1:400, Fujifilm Wako Chemicals, Richmond, VA, #019-19741) and to CD68 (1:500, Biorad, Hercules, CA, #MCA341R). DAPI was used to counterstain nuclei.

Vectashield mounted samples were imaged using a Leica TSP5 laser scanning confocal microscopy system. X-Y stacks were collapsed to yield representative maximal projections for image quantification.

Quantification of images was performed manually aided by ImageJ software. To quantify numbers of CD68 or Iba-1 positive microglia in tissue sections, counts from 3 sections for each tissue were averaged to obtain the number of microglia per 350 μm of retina, and a minimum of 3 tissues from 3 animals per sample type were analyzed. To quantify the number of photoreceptor nuclei rows in tissue sections, in each section the number of photoreceptor rows was counted in 3 regions of the image (left, central, and right) and averaged.

### SDS PAGE and Immunoblotting

Dissected single tissues were lysed in HNTG buffer (50 mM HEPES, 150 mM NaCl, 1% Triton-X100, 10% glycerol, pH 7.5) freshly supplemented with protease inhibitor cocktail. Proteins from cleared lysates were separated by standard SDS-PAGE and transferred to nitrocellulose membranes, Blots were blocked in 10% non-fat milk powder in TBS before incubation with primary and appropriate HRP-conjugated secondary antibodies, and enhanced chemiluminescence digital detection by a KwikQuant Imager (Kindle Biosciences, Greenwich, CT). Band densities were quantified using the KwikQuant imaging software. Primary antibodies used were to CD68 (1:1,000, Biorad #MCA341R), Iba-1 (1:2,000, Fujifilm Wako Chemicals, #016-20001), PSD95 (1:3,000, Cell Signaling, Danvers, MA, #3450), and α-tubulin (1:3,000, Cell Signaling, #9099). For all immunoblotting experiments, tissues from 2 different rats of each sample group were directly compared in 3 independent experiments. Tubulin reprobing of membranes was used to control for sample load.

### Statistical Analysis

All data were collected from at least three independent experiments. The means and standard deviations were calculated for each comparison group. Comparisons between two groups were performed using the Student's two-tailed *t*-test. Comparisons between three or more groups were performed using one-way or two-way ANOVA as appropriate, with Tukey's *post-hoc* test for comparison of two groups within multiple groups. *P* values below 0.05 were considered statistically significant for all experiments.

## Results

### The Pro-inflammatory Cytokine CCL5 and Microglia Activation Marker Iba-1 Are Elevated Even Prior to Eye Opening in RCS Rat Retina

**Figure 1:**
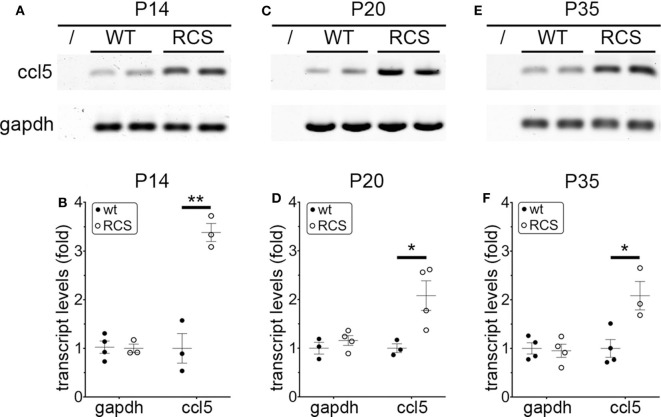
CCL5 mRNA overexpression begins before eye opening in RCS rat retina. Representative RT-PCR products for the cytokine CCL5 and the housekeeping gene GAPDH are shown in duplicate at P14 **(A)**, P20 **(C)**, and P35 **(E)** for WT and RCS retina tissues harvested, respectively. Lanes represent contamination controls with water as template. Ages are shown in figure panels with quantifications shown in **(B,D,F)** as mean ± s.e.m., *n* = 4 biological samples from 3 individual rats. Levels are shown relative to WT. Data were analyzed by Student *t*-test; ** indicates *p* < 0.01, * indicates *p* < 0.05.

**Figure 2:**
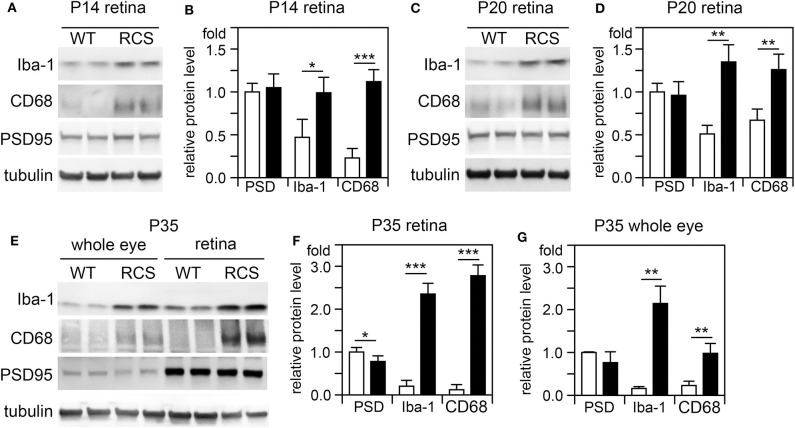
Microglia activation marker proteins increase in RCS retina by P14, before eye opening. Tissues dissected at ages indicated were analyzed by SDS-PAGE/immunoblotting. **(A,C,E)** Representative immunoblots of duplicate samples from different retina **(A,C,E)** or whole eye tissue following removal of the lens **(E)** of ages as indicated tested for Iba-1, CD68, PSD95, and tubulin as loading control. **(B,D,F,G)** Quantification of PSD95 (PSD), Iba-1, and CD68 levels from immunoblots as in **(A,C,E)**. Bars show mean ± SD, *n* = 3 biological samples from 3 individual rats. Protein levels are shown relative to tubulin content of the same sample and relative to level of WT PSD95, which was set as 1. Data were analyzed by Student *t*-test; *** indicates *p* < 0.001, ** indicates *p* < 0.01, * indicates *p* < 0.05.

Cytokine secretion is one of the first indications of tissue inflammation. Once secreted these small molecules serve to attract inflammatory cells expressing specific cytokine receptors, causing migration to inflammatory sites. As reported earlier by others, inflammatory cytokines in *mertk*^−/−^ mouse retina are present at P35, a late disease stage (Supplementary Figure 1A). Strikingly, we found levels of mRNA for CCL5 (Chemokine C-C motif ligand 5) already elevated at P14, the day of or after eye opening of our mouse strains (Supplementary Figure 1B). CCL5 is a pro-inflammatory cytokine whose elevation has been linked to retinal stress associated with glaucoma and age-related macular degeneration (37, 38). To determine whether this observation was characteristic for MerTK deficient retina, we next tested CCL5 levels in RCS rat retina. Indeed, we found that CCL5 is also significantly elevated in RCS retina at P14, one day prior to eye opening in our rats (Figure 1). Compared to WT retina, RCS retina contained 3.4-fold higher levels of CCL5 transcripts at P14 (Figures 1A,B) as compared to ~2-fold higher levels at P20 (Figures 1C,D), and P35 (Figures 1E,F), the previously recognized early and late stage of RCS retina degeneration, respectively. This was somewhat unexpected because diurnal photoreceptor outer segment renewal is thought to commence in rodent retina only after eye opening, and thus any process occurring prior is unlikely to be a consequence of its deficiency due to the RPE phagocytosis defect. Unlike CCL5 transcripts, levels of GAPDH transcripts were the same in WT and age-matched RCS retina indicating that the change in CCL5 was not due to global change in transcription (Figure 1). Motivated by prior reports on microglia activation in RCS rat retina, we next compared levels of the microglia markers Iba-1 and CD68 between RCS and WT rat dissected neural retina. Resident tissue microglia upregulate basal levels of Iba-1 upon activation and this change correlates with their migration to sites of tissue injury (39, 40). CD68 is expressed only in activated retinal microglia (41). Figure 2 shows that already at P14, Iba-1 and CD68 protein levels in RCS retina are 2.2-fold and 4.9-fold higher than levels in WT rat retina, respectively (Figures 2A,B). By P20, Iba-1 levels had increased to 2.6-fold of WT levels, while CD68 levels were 1.9-fold elevated over WT (Figures 2C,D). We noted that CD68 levels relative to tubulin loading control increased little in RCS retina from P14 to P20, while CD68 in WT retina rose 2.9-fold. By P35, Iba-1 and CD68 levels in RCS retina were 12-fold and 23-fold of WT levels, respectively (Figures 2E,F). Differences in microglia marker content between RCS and WT tissues were more pronounced in dissected retina samples than in whole eye samples indicating a primary role for retinal rather than choroidal microglia (Figure 2, compare F and G). As control for global neural tissue changes, we also measured levels of the synaptic marker PSD95 at all ages. We found no differences between RCS and WT PSD95 at P14 and P20 and only modest decline at P35, which did not reach statistical significance (Figure 2, panels and bars as indicated). Up to P35, tubulin levels were identical in age-matched RCS and WT rat retina confirming that overall retina cell loss is negligible by this age. All protein levels were thus quantified relative to tubulin levels in the same sample and development to account for differences in tissue yield during dissections. Altogether, these results suggested that inflammatory signaling and responses by microglia cells in RCS rat retina commence before eye opening.

### RPE Phagocytosis in P14 Wild-Type Rat Retina Just Prior to Eye Opening Is Only Partially Active

**Figure 3:**
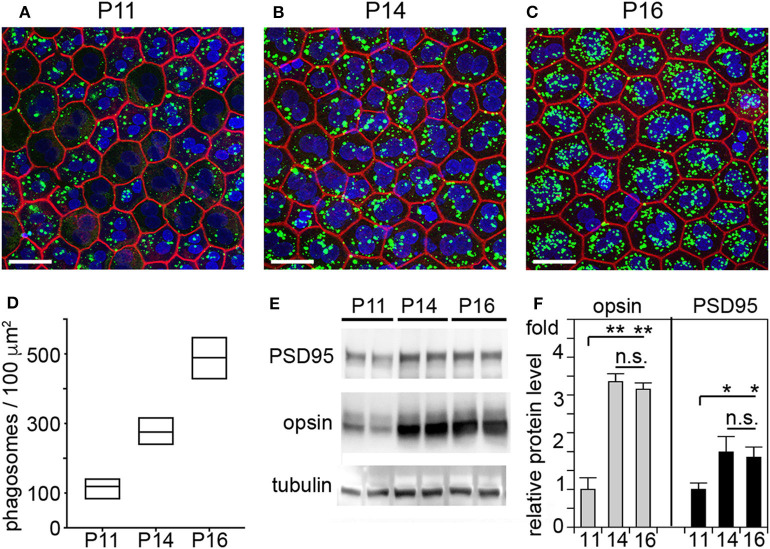
RPE phagocytosis is fully active in WT rat retina only at P16, after eye opening. WT rats of defined age as indicated were sacrificed 1 h after light onset followed by immunofluorescence labeling of posterior eyecup whole mounts **(A–D)** and protein extraction and immunoblotting **(E,F)** of contralateral eyes of same animal. **(A–C)** Representative fields show whole mount samples at ages indicated co-stained for rhodopsin positive phagosomes (green), F-actin (red) and nuclei (blue). Scale bar, 25 μm. **(D)** Quantification of phagosomes from images as in **(A)**. Box graphs show line corresponding to the mean ± min and max values, *n* = 3 biological samples from 3 rats. Data were analyzed by 1-way ANOVA, ** indicates *p* < 0.01; * indicates *p* < 0.05. **(E)** Representative immunoblots of duplicate samples from different WT rat whole eyes of ages as indicated tested for rhodopsin, PSD95, and tubulin as indicated. One membrane sequentially probed for the different proteins is shown. **(F)** Quantification of rhodopsin (opsin) and PSD95 levels from immunoblots as in **(C)**. Bars show mean ± SD, *n* = 3 biological samples from 3 individual rats. Protein levels are shown relative to tubulin content of the same sample and relative to level of PSD95 and rhodopsin at P11, which was set as 1. Data were analyzed by ANOVA, ** indicates *p* < 0.01; * indicates *p* < 0.05.

It has long been assumed that outer segment renewal in rodents starts after eye opening. The WT rats tested in our study fully open eyes at P15. Here, we measured outer segment phagosome load of the RPE *in situ* as quantitative assessment of outer segment renewal. Figure 3 shows rhodopsin-positive phagosomes in WT rat RPE and their quantification 1 h after light onset, when phagosome load is at its peak. Compared to P11, RPE phagocytosis at P14 one day prior to eye opening is about 2-fold more active (Figures 3A,B). However, RPE phagosome load is still 44% lower at P14 compared to P16, one day after eye opening (Figures 3A,B). Notably, we found no difference in total levels of retinal or RPE marker proteins including the rod outer segment marker rhodopsin between P14 and P16 but less rhodopsin at P11 in WT rat eyes (Figures 3C,D). These results suggest that outer segment formation is complete by P14, 1 day before eye opening, but outer segment renewal is not yet fully active. Eye opening of our RCS rats between P15 and P16 implies that at P14 there is little to no debris accumulation that could distress photoreceptors, and that therefore cytokine elevation in P14 RCS retina is highly unlikely a consequence of lack of RPE phagocytosis.

### Microglia Migrate to Reach the Photoreceptor Layer of RCS Rat Retina as Early as P20

**Figure 4:**
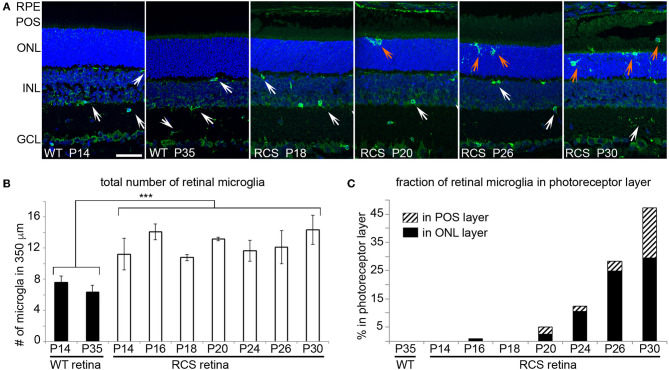
Iba-1 positive microglia migrate from the inner retina to the photoreceptor layer of RCS retina leading to significantly increased in outer retina microglia by P20. Microglia in WT and RCS rat retina cross sections were labeled with Iba-1 antibody (shown in green) and examined by immunofluorescence microscopy. Nuclei counterstain is shown in blue. **(A)** Representative microscopy fields show retina of WT or RCS rats at ages as indicated in the panels. Select inner retina microglia are indicated by white arrows, while outer retina microglia are indicated by orange arrows. In WT retina, microglia were only observed in the inner retina regardless of age. Outer nuclear layer (ONL), photoreceptor outer segment layer (POS). Scale bar, 40 μm. **(B)** Quantification of the total number of Iba-1 positive microglia in the retina shows increased numbers of microglia in RCS retina by P14 and no further change with age (mean ± SD, *n* = 3 biological replicates, ***p < 0.001, ANOVA). **(C)** Quantification of fraction of Iba-1 positive microglia in the outer retina separated by ONL and POS layer as indicated shows increasing migration to the ONL and POS layers of the retina with age.

We next determined the localization of Iba-1 positive retinal microglia in RCS rat retina with increasing age. As expected, WT retina harbored microglia only in the inner retina regardless of age (Figure 4A, WT panels as indicated). In contrast, all RCS retina samples tested showed microglia in the inner as well as the outer retina starting at P20 (Figure 4A, RCS panels as indicated). Quantification of Iba-1 positive cells over the entire retina showed that the total number of Iba-1 positive cells in RCS rat retina did not change from P14 to P30, although it was 1.8-fold higher than cell numbers in WT retina as expected from prior studies on rats aged P21 and up (34) (Figure 4B). Additionally, the fraction of Iba-1 positive cells in the outer retina starting at P20 increased dramatically with age (Figure 4C). These results suggest that microglia numbers are already elevated in RCS rat retina before onset of retinal degeneration and prior to eye opening. Moreover, increase in outer retina microglia in RCS rats likely arises from migration of resident microglia rather than cell proliferation or infiltration.

### Suppression of Microglia by Tamoxifen Eye Drops Mitigates Photoreceptor Death

**Figure 5:**
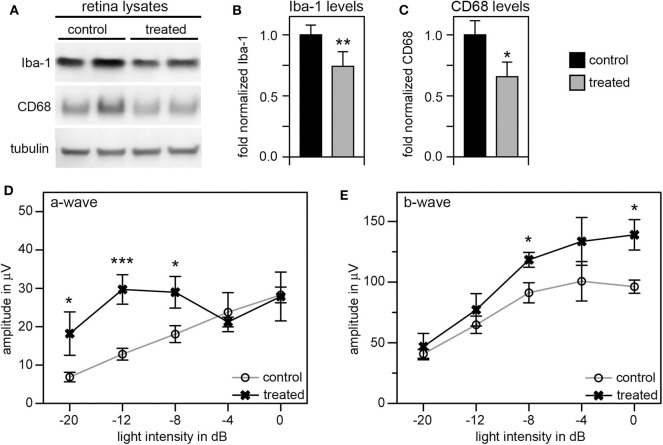
Tamoxifen eye drops yield modest but significant decrease in retinal Iba-1 levels and preservation of retinal function of RCS rat retina. RCS rats received tmx eye drops three times daily from P10 to P40 with examination by scotopic ERG at P33 and tissue harvest for immunoblotting analysis at P40. **(A)** A representative immunoblot is shown with two biological replicates each of retina extracts from control and treated RCS rats. The same membrane was probed for Iba-1, CD68, and for tubulin, as loading control. **(B,C)** Bar graphs show quantification of experiments as in **(A)** with levels of Iba-1 **(B)** and CD68 **(C)** in each sample set relative to levels of tubulin before comparing control and treated samples. Normalized Iba-1 and CD68 levels in control samples were set as 1 (mean ± SD, *n* = 4 biological replicates; *p ≤ 0.05, **p ≤ 0.01; Student's *t*-test). **(D,E)** Graphs show average a-wave and b-wave amplitudes in response to light flashes of increasing intensity as indicated (mean ± s.e.m., n = 5 biological replicates, (*p ≤ 0.05; ***p < 0.001, ANOVA). Open circles: RCS rats treated with oil eye drops (control); X: littermate RCS rats treated with tmx eye drops (treated).

Given these unexpectedly early changes of microglia in RCS retina, we decided to continue our studies by using tamoxifen (tmx) to inactivate microglia starting in rat pups at P10. Tmx is an estrogen receptor agonist long used to treat breast cancer in human patients (42). In experimental animals, tmx is commonly used to control gene expression via engineered tmx-inducible promoters (43). Systemic application, via intraperitoneal injection or oral supplementation, and topical administration via creams and eye drops have been successfully used in published studies (35, 43, 44). Schlecht and colleagues showed that tmx administration as eye drops from P8 through P12 yields effects on outer retinal cells indicating that topical tmx penetrates eye tissues reaching effective concentration even in the posterior retina (35). Notably, such topical administration of tmx to the eye (45) does not affect retinal morphology even though tmx can be toxic to the retina (46). Even more recently, Wang and colleagues showed that tmx provided as dietary supplement reduced the extent of photoreceptor degeneration caused by light damage by reducing the number of activated microglia (47). The mechanism used by tmx to deplete activated microglia is still unknown, but it appears to be independent of its effects on estrogen receptors (47). To suppress microglia activity starting at pre-weaning age we applied tmx as eye drops in RCS rats three times a day from P10. At P33 we recorded scotopic ERGs to assess photoreceptor functions, and at P40 we collected retina tissues to quantify levels of Iba-1 and CD68 proteins by immunoblotting. These experiments yielded a modest but significant reduction by about one third in both of these microglia markers in tmx-treated RCS rat eyes compared to control littermates that received eye drops with corn oil solvent only (Figures 5A–C). Moreover, tmx-treated rats at P33 showed significantly better light responses than control RCS rats at lower flash intensities eliciting rod activation only (−20dB to −8dB). At high flash intensities that excite both rods and cones there was no difference in retinal responses based on treatment (−4dB and 0dB) (Figure 5D). The b-wave indicative of activities of second order retinal neurons was also improved by tmx eye drop treatment (Figure 5E). RCS rats with ongoing retinal degeneration do not show normal retinal activity in ERG recordings with consistent increases in a- and b-wave amplitudes in response to light flashes of increasing intensity (23). Recording at P33 specifically in our study we observed flat or even moderately declining a- and b-wave amplitudes at highest flash intensities applied indicative of dysfunction of photoreceptors at this age. Nonetheless, tmx treatment modestly improved retinal function.

### Adding Tamoxifen Diet to Tamoxifen Eye Drops Treatment Further Suppresses Microglia but Is Toxic

**Figure 6:**
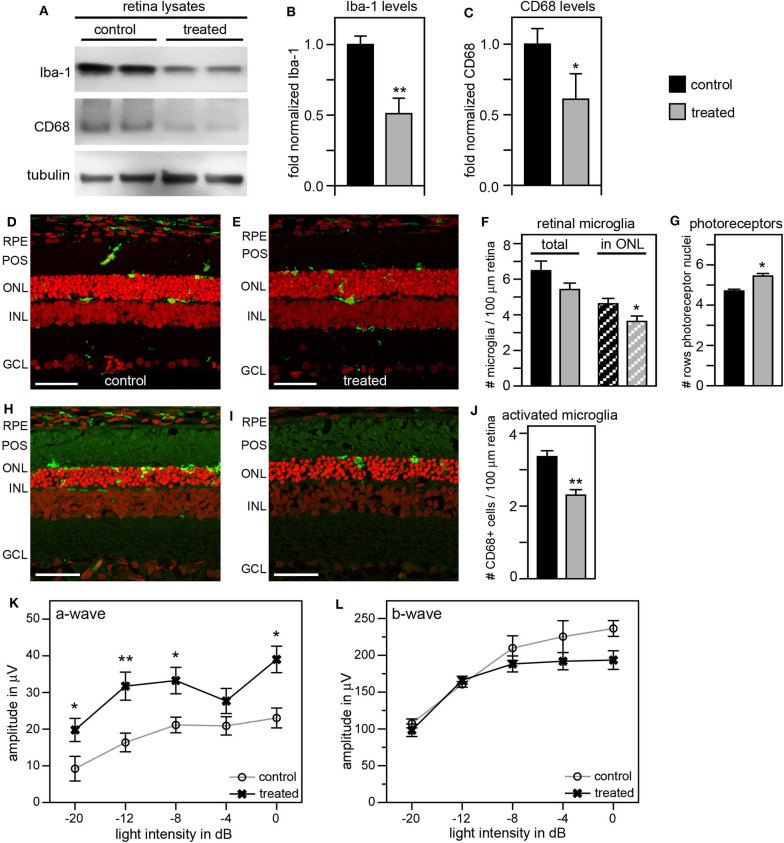
Tamoxifen provided both as eye drops and as dietary supplement yields significant preservation of retinal function and decrease in retinal Iba-1 levels in RCS rats. RCS rats received tmx eye drops three times daily from P10 to P40 and standard chow enriched with tmx starting at P19 with examination by scotopic ERG at P33 and tissue harvest for immunoblotting analysis at P40. **(A)** A representative immunoblot is shown comparing retina extracts from control and treated RCS rats. The same membrane was probed for Iba-1, CD68, and for tubulin as loading control. **(B,C)** Bar graphs show quantification of experiments as in **(A)** with levels of Iba-1 **(B)** and CD68 **(C)** in each sample set relative to levels of tubulin before comparing control and treated samples. Normalized Iba-1 and CD68 levels in control samples were set as 1 (mean ± SD, *n* = 4 biological replicates; *p ≤ 0.05, **p ≤ 0.01, Student's *t*-test). **(D,E)** Representative micrographs of retina cross sections labeled with Iba-1 antibody to indicate microglia (green) and nuclei counterstain (red). The same regions of the retina were imaged to directly compare retina of control and treated RCS rats. Scale bars, 40 μm. **(F)** Bar graph shows quantification of total retinal or photoreceptor layer microglia as indicated (mean ± s.e.m., *n* = 5 biological replicates; *p ≤ 0.05, Student's *t*-test). **(G)** Bar graph shows number of rows of photoreceptor nuclei in the outer nuclear layer (mean ± s.e.m., *n* = 5–7 biological replicates; *p ≤ 0.05, Student's *t*-test). **(H,I)** Representative micrographs of retina cross sections labeled with CD68 antibody to indicate activated microglia (green) and nuclei counterstain (red). The same regions of the retina were imaged to directly compare retina of control and treated RCS rats. Scale bars, 40 μm. **(J)** Bar graph shows quantification of activated retinal microglia as indicated (mean ± s.e.m., *n* = 4 biological replicates; **p ≤ 0.005; Student's *t*-test). **(K,L)** Graphs show average a-wave and b-wave amplitudes in response to light flashes of increasing intensity as indicated (mean ± s.e.m., *n* = 4–9 biological replicates, *p ≤ 0.05; **p < 0.005, ANOVA). Open circles: RCS rats treated with oil eye drops and fed standard chow (control); X: littermate RCS rats treated with tmx eye drops and fed tmx chow (treated).

To improve the retinal degeneration delay effects observed from tmx eye drops alone, we modified the treatment by adding tmx-supplemented diet starting at weaning at P19. This combined tmx eye drop/diet strategy reduced both Iba-1 and CD68 in treated RCS rat retina by ~50 and 40%, respectively, compared to control siblings receiving neither topical nor systemic tmx (Figures 6A–C). As complement, we performed immunofluorescence microscopy analysis of retina tissue sections. We observed fewer Iba-1 positive microglia specifically in the outer nuclear layer of the retina of tmx treated RCS rats compared to control littermates (Figures 6D–F). Moreover, the retina of tmx-treated RCS rats showed one additional row of photoreceptor nuclei suggesting modestly improved retention of photoreceptor cells (Figures 6D,E,G). We also found fewer CD68 positive, activated microglia, in treated rats compared to control rats (Figures 6H–J). Scotopic ERGs at P33 showed an improvement of the photoreceptor response at all light flash intensities (Figure 6K). However, b-wave amplitudes did not differ between tmx-treated and control animals (Figure 6L). Moreover, animals treated with tmx diet were smaller, more sensitive to the anesthetic, and overall less healthful than control siblings (data not shown). Taken together, we conclude from these observations that combining topical and systemic tmx treatments is more effective in inactivating retinal microglia than topical treatment alone. However, considerable systemic tmx toxicity renders such dual application unsuitable for further research let alone translation.

### Tamoxifen Eye Drops Combined With Intravitreal and Systemic Injection of Liposomal Clodronate Reduces Photoreceptor Death by Suppressing Microglia Activation

**Figure 7:**
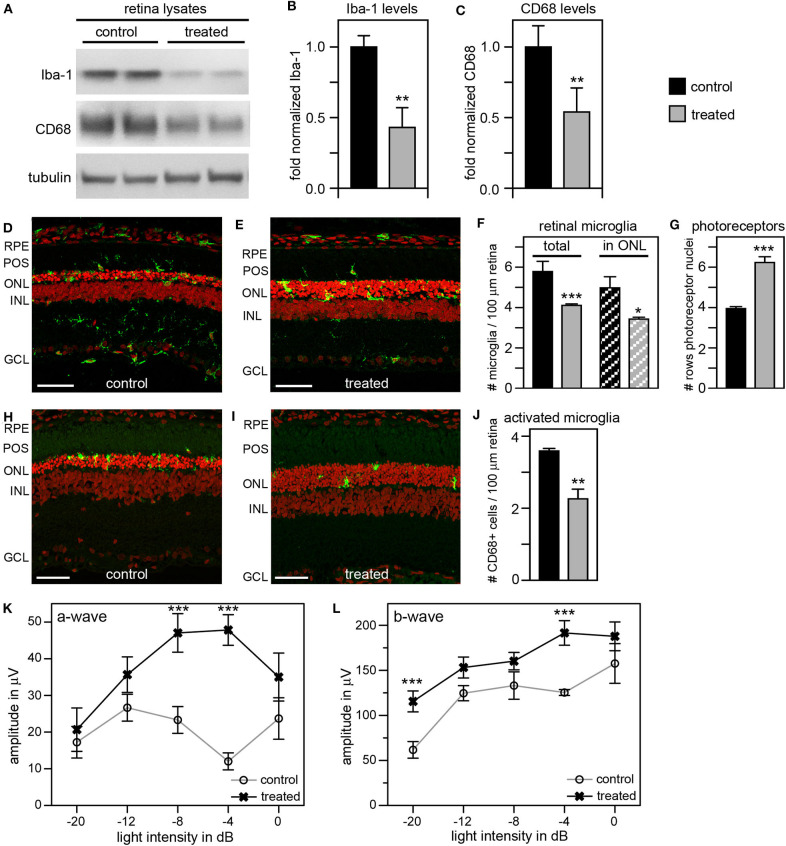
A combination treatment of tamoxifen eye drops and intraperitoneal liposomal clodronate yields robust preservation of retinal function, decreases retinal Iba-1 levels and microglia redistribution to the outer retina and preserves photoreceptors. RCS rats received tmx eye drops three times daily from P10 to P40, intravitreal injection with LC at eye opening (P16-P17) and intraperitoneal injection at P13 and every 7 days thereafter with examination by scotopic ERG at P33 and tissue harvest for immunoblotting and histology analyses at P40. **(A)** A representative immunoblot is shown comparing retina extracts from control and treated RCS rats. The same membrane was probed for Iba-1, CD68, and for tubulin as loading control. **(B,C)** Bar graphs show quantification of experiments as in **(A)** with levels of Iba-1 **(B)** and CD68 **(C)** in each sample set relative to levels of tubulin before comparing control and treated samples. Normalized Iba-1 and CD68 levels in control samples were set as 1 (mean ± SD, *n* = 4 biological replicates; **p ≤ 0.01, Student's *t*-test). **(D,E)** Representative micrographs of retina cross sections labeled with Iba-1 antibody to indicate microglia (green) and nuclei counterstain (red). The same regions of the retina were imaged to directly compare retina of control and treated RCS rats. Scale bars, 40 μm. **(F)** Bar graph shows quantification of total retinal or photoreceptor layer microglia as indicated (mean ± s.e.m., n = 5 biological replicates; *p ≤ 0.05; ***p ≤ 0.001, Student's *t*-test). **(G)** Bar graph shows number of rows of photoreceptor nuclei in the outer nuclear layer (mean ± s.e.m., *n* = 5–7 biological replicates; ***p ≤ 0.001, Student's *t*-test). **(H,I)** Representative micrographs of retina cross sections labeled with CD68 antibody to indicate activated microglia (green) and nuclei counterstain (red). The same regions of the retina were imaged to directly compare retina of control and treated RCS rats. Scale bars, 40 μm. **(J)** Bar graph shows quantification of activated retinal microglia as indicated (mean ± s.e.m., *n* = 4 biological replicates; **p ≤ 0.005; Student's *t*-test). **(K,L)** Graphs show average a-wave and b-wave amplitudes in response to light flashes of increasing intensity as indicated (mean ± s.e.m., *n* = 4–9 biological replicates, ***p ≤ 0.001, ANOVA). Open circles, RCS rats treated with oil eye drops (control); X, littermate RCS rats treated with tmx eye drops and LC (treated).

As an alternate strategy to increase efficiency of microglia inhibition, we tested the addition of local and systemic administration of liposomal clodronate (LC) to our previous tmx eye drop treatment. LC is selectively taken up by phagocytic cells triggering their apoptotic pathway (48, 49). Intravitreal LC injection has been shown to successfully deplete retinal microglia in several species including rats (50–52). In a pilot experiment, we confirmed that a one-time intravitreal injection one day after eye opening reduces the number of Iba-1 positive retinal microglia in RCS rats (Supplementary Figure 2). To increase the efficacy of our anti microglia treatment, we then combined inactivation with depletion of microglia by applying tmx eye drops starting at P10 as in our previous experiments and adding a one-time intravitreal LC injection the day after eye opening. To suppress replenishment of retinal microglia we additionally applied LC intraperitoneally every 7 days starting at P13. Indeed, Figure 7 shows that this combination treatment was more effective than tmx eye drops alone or even tmx eye drops with the toxic tmx food. Immunoblotting showed reduction of retinal Iba-1 levels in treated animals by about 60% and of CD68 by about 50% compared to protein levels in sibling controls, indicating a greater reduction of microglia and their activation in RCS rats treated with the combined treatment as compared to rats treated with tmx alone (Figures 7A–C). Moreover, ~30% fewer microglia localized to the photoreceptor layer of the retina (the outer nuclear layer) suggesting impaired microglia migration in response to tmx/LC treatment and the number of total Iba-1 positive retinal microglia was similarly reduced (Figures 7D–F). The combination treatment directly benefitted photoreceptor viability preserving more than two additional rows of photoreceptor nuclei in LC/tmx treated retina by P40 compared to control sibling retina (Figures 7D,E,G). Matching the immunoblotting results, immunofluorescence microscopy of CD68 showed a significant reduction of about 40% in activated microglia in retina from treated rats (Figures 7H–J). Finally, scotopic ERGs revealed significantly higher a-wave and b-wave amplitudes in treated RCS rats confirming improved retinal functionality (Figures 7K,L). Importantly, the combination treatment did not show obvious systemic toxicity. Altogether, these results suggest that a combination treatment of tmx eye drops and LC injections is more efficient in suppressing microglia than tmx eye drops alone. Our results imply that reducing microglia activity delays degeneration of photoreceptors in mutMerTK-RP.

## Discussion

In this study, we investigated the age of onset and effects of early treatment of inflammatory changes in animal models lacking the phagocytosis receptor MerTK. The MerTK-deficient mice and rats we studied exhibit early onset fast progressing retinal degeneration like human patients with mutMerTK-RP. We found pro-inflammatory cytokines, elevated levels of retinal microglia and microglia activation markers prior to eye opening. It has long been assumed (but never directly tested as far as we are aware) that eye opening marks the beginning of life-long diurnal outer segment renewal including MerTK-dependent RPE phagocytosis. Indeed, our results confirm that there is only limited RPE phagocytosis of spent outer segment fragments before eye opening in WT rat retina whose RPE cells require MerTK for daily engulfment of spent photoreceptor outer segment tips. These results suggest that inflammatory activation prior to eye opening in MerTK-deficient retina is highly unlikely a mere consequence of the accumulation of outer segment debris in the subretinal space due to phagocytosis-defective RPE.

In both mouse and rat models of MerTK deficiency, we found expression of the chemoattractant cytokine CCL5 (and others) significantly and robustly elevated at P14 prior to eye opening and before photoreceptor debris buildup due to lack of MerTK-mediated RPE phagocytosis. The cells producing the cytokines remain to be specified. RPE *in vivo* may produce CCL5 and other cytokines in culture for instance in response to viral infection (53). Given that we studied dissected retina samples from which the RPE and posterior eyecup was removed, it is however likely that retinal cells not RPE or choroidal cells generate these transcripts. The robust and consistent level of CCL5 mRNA in our retina extracts renders contaminating RPE unlikely as major source.

Likely as response to elevated cytokines, we observed microglia relocalization to the outer retina as early as P20. In elegant experiments, Kohno et al. recently discriminated between retinal microglia and invading macrophages in *mertk*^−/−^ mice by Cx3cr1 and Ccr2 promotor labeling, respectively. They found invading macrophages in *mertk*^−/−^ retina only at ages of 6 weeks and above (32). This suggests that retinal microglia are the Iba1-positive cells population we observed in our MerTK-deficient RCS rats at ages up to P40. In agreement with previous studies that mostly focused on RCS rat retina in mid to late stage retinal degeneration at P33 and older (31, 34), we saw increased numbers of microglia in RCS rat retina as compared to WT retina at all ages tested including before eye opening. The total number of microglia did not increase, suggesting that the increased number of microglia observed in the outer retina from P21 was solely due to migration and not proliferation. In agreement, Di Pierdomenico et al. showed proliferation of retinal microglia in RCS retina at P45 but not at P33 (34).

Inflammation has been reported mostly at advanced stages of RP in retina that has already partly degenerated and in which retinal cells continue to die (31, 33, 54–56). However, precedent exists that microglia activation may occur early, preceding any retinal cell death, and contribute to degenerative processes (57). Microglia may have protective roles (24, 58) or promote photoreceptor cell death increasing the severity of retinal degeneration (29, 40, 59). To investigate the relevance of the early microglia activation for RCS rat retinal degeneration, we inhibited microglia using two experimental approaches, which we begun 4 days before eye opening. First, we inhibited microglia with tmx shown previously to inactivate retinal microglia in adult mice (47). Application of tmx as eye drops alone yielded a small but significant reduction in microglia activation and marginally better photoreceptor light responses at P33. To enhance efficacy and to prevent possible systemic replenishment of microglia to tmx eye drop-treated retina, we next added feeding animals tmx-supplemented diet to the tmx eye drop treatment. While this dual treatment showed improved efficacy in the retina we observed significant toxicity of the tmx diet resulting in reduced body weight and viability. Therefore, we instead complemented the tmx eye drop administration with a well-established alternative approach depleting microglia with liposomal clodronate, which poisons microglia. Combined local treatment of tmx eye drops and one-time intravitreal injection of LC with weekly systemic LC injections further increased microglia depletion compared to eye drops alone with no apparent toxicity. Taken together, these experiments demonstrate that early microglia inhibition is effective in delaying retinal degeneration due to MerTK deficiency. Moreover, the extent of microglia inhibition directly correlates with improved survival of photoreceptors and preservation of photoreceptors function. Finally, our results indicate that microglia driven processes aggravate retinal degeneration due to MerTK deficiency rather than playing a protective role.

More experiments are needed to unravel how microglia aggravate retinal degeneration due to deficiency in the engulfment receptor MerTK. In brain, MerTK deficiency impairs efferocytosis activity of microglia and macrophages (60, 61). In the retina of the rd10 mouse model of RP, microglia phagocytose living photoreceptors increasing the rate of photoreceptor loss (59). It will be important to test microglia phagocytosis in RCS retina in the future. It is important to note however that the genetic defect in the rd10 RP model is in *pde6b*, which encodes a photoreceptor-specific protein involved in phototransduction. In contrast, MerTK is a phagocytosis receptor expressed not by photoreceptors but by the adjacent RPE, which relies on it for daily clearance of spent outer segment tips as part of outer segment renewal and possibly by other phagocytic cells in the retina. We did not find reports showing MerTK protein expression in mouse or rat retinal microglia *in situ*. With MerTK antibodies recognizing rat MerTK in tissue sections unavailable, we tested MerTK localization in WT mouse retina. Supplementary Figure 3 shows abundant labeling by a well-established anti-mouse MerTK antibody of WT mouse RPE but little labeling in neural retina and no obvious overlap with Iba-1 microglia marker. Thus, MerTK protein is unlikely to be abundant in resting retinal microglia, and its expression may be upregulated in retinal disease such as rd10 RP to enhance microglia phagocytosis of distressed photoreceptors. If the aggravating role of retinal microglia in MerTK-deficient RP also involves photoreceptor phagocytosis it will indicate that retinal microglia perform clearance phagocytosis using a MerTK-independent pathway with an alternative engulfment receptor. Indeed, numerous engulfment receptors relevant to efferocytosis are known (62). Moreover, increased staining of complement factor C1q has been reported in RCS retina in the OPL where microglia may phagocytose synaptic components (63). The same study found that elimination of retinal microglia by systemic treatment with a CSF1 receptor inhibitor had no effect on photoreceptor light responses (and worsened inner retinal responses), although drug administration via dietary supplementation only started at P15 and may not have been effective immediately in pre-weaning rats. As dead cells do not permanently accumulate in advanced stages of retinal degeneration in RCS retina clearance phagocytosis is likely to take place, although the specific phagocytic cells responsible and whether and when they enter the retina is unknown. Finally, microglia in MerTK-deficient retina may impair photoreceptor function and viability through activities that do not require them to be phagocytic. Distinguishing between these intriguing possibilities will be the subject of further investigation.

As of now, there is no cure for any form of RP. However, translational approaches including clinical trials are in development or underway (64, 65). Yet, treatments in development such as gene therapy or RPE replacement will be successful only if there is significant retention of retinal cells and architecture whose functionality and longevity can be rescued by treatment. Delaying the exceptionally early onset and rapid progression to irreversible retinal cell death that is characteristic for mutMerTK-RP will increase the window of opportunity during which advanced treatments such as gene therapy will be effective. As our results show a role for early inflammation before the phagocytic defect of the RPE harms photoreceptors, it is tempting to speculate that other forms of RP may benefit from microglia inactivation at very early stages of disease prior to overt retinal dysfunction and degeneration.

Altogether, our results show that lack of MerTK activates inflammatory mechanisms causing retinal microglia to harm photoreceptors prior to onset of fully active daily outer segment renewal. These processes are thus independent of accumulation of subretinal outer segment debris that results from engulfment failure of MerTK-deficient RPE. In addition to orchestrating debris engulfment, MerTK signaling is complex and has been shown to contribute to cell differentiation and homeostasis (66). Our results let us hypothesize that cells lacking MerTK in the mutMerTK retina elicit pro-inflammatory signaling which in turn activates microglia. Additional studies will be needed to determine whether the triggering cell type is the RPE, which may utilize MerTK signaling during normal development and differentiation. Distressed MerTK-deficient RPE cells may communicate with cells in the retina via additional cytokines or other signaling mediators causing these secondary cells to upregulate the cytokines we found elevated. Identifying the primary and secondary cell types and stimulus mechanism for such inflammatory pathways will be a subject of future studies and may identify cellular molecular targets for therapies aiming to delay onset of the aggressive forms of retinitis pigmentosa associated with defective MerTK.

## Data Availability Statement

The datasets generated for this study are available on request to the corresponding author.

## Ethics Statement

All procedures involving animals were performed according to the ARVO Guide for Use of Animals in Vision Research and the *Guide for the Care and Use of Laboratory Animals* (NIH, 8th edition) and approved by the Institutional Animal Care and Use Committee of Fordham University.

## Author Contributions

This study was designed by DL, FM, and SF. DL, FM, and SF performed experiments, analyzed, and interpreted results. DL and FM wrote the manuscript with input from SF. All authors read, commented, and approved of the final manuscript.

## Conflict of Interest

The authors declare that the research was conducted in the absence of any commercial or financial relationships that could be construed as a potential conflict of interest.

## References

[1] Ferrari S Di Iorio E Barbaro V Ponzin D Sorrentino FS Parmeggiani F. Retinitis pigmentosa: genes and disease mechanisms. Curr Genomics. (2011) 12:238–49. 10.2174/138920211795860107 22131869 PMC3131731

[2] Daiger SP Sullivan LS Bowne SJ Genes and mutations causing retinitis pigmentosa. Clin Genet. (2013) 84:132–41. 10.1111/cge.12203 23701314 PMC3856531

[3] Dias MF Joo K Kemp JA Fialho SL da Silva Cunha AJr Woo SJ. Molecular genetics and emerging therapies for retinitis pigmentosa: Basic research and clinical perspectives. Prog Retin Eye Res. (2018) 63:107–31. 10.1016/j.preteyeres.2017.10.004 29097191

[4] Gal A Li Y Thompson DA Weir J Orth U Jacobson SG. Mutations in MERTK, the human orthologue of the RCS rat retinal dystrophy gene, cause retinitis pigmentosa. Nat Genet. (2000) 26:270–1. 10.1038/81555 11062461

[5] McHenry CL Liu Y Feng W Nair AR Feathers KL Ding X. MERTK arginine-844-cysteine in a patient with severe rod-cone dystrophy: loss of mutant protein function in transfected cells. Invest Ophthalmol Vis Sci. (2004) 45:1456–63. 10.1167/iovs.03-0909 15111602

[6] Tschernutter M Jenkins SA Waseem NH Saihan Z Holder GE Bird AC. Clinical characterisation of a family with retinal dystrophy caused by mutation in the Mertk gene. Br J Ophthalmol. (2006) 90:718–23. 10.1136/bjo.2005.084897 16714263 PMC1860205

[7] Mackay DS Henderson RH Sergouniotis PI Li Z Moradi P Holder GE. Novel mutations in MERTK associated with childhood onset rod-cone dystrophy. Mol Vis. (2010) 16:369–77. 20300561 PMC2838735

[8] Ostergaard E Duno M Batbayli M Vilhelmsen K Rosenberg T. A novel MERTK deletion is a common founder mutation in the Faroe Islands and is responsible for a high proportion of retinitis pigmentosa cases. Mol Vis. (2011) 17:1485–92. 21677792 PMC3110495

[9] Ksantini M Lafont E Bocquet B Meunier I Hamel CP. Homozygous mutation in MERTK causes severe autosomal recessive retinitis pigmentosa. Eur J Ophthalmol. (2012) 22:647–53. 10.5301/ejo.5000096 22180149

[10] Al-Khersan H Shah KP Jung SC Rodriguez A Madduri RK Grassi MA. A novel MERTK mutation causing retinitis pigmentosa. Graefes Arch Clin Exp Ophthalmol. (2017) 255:1613–9. 10.1007/s00417-017-3679-9 28462455 PMC5542860

[11] Dowling JE Sidman RL. Inherited retinal dystrophy in the rat. J Cell Biol. (1962) 14:73–109. 10.1083/jcb.14.1.73 13887627 PMC2106090

[12] D'Cruz PM Yasumura D Weir J Matthes MT Abderrahim H LaVail MM. Mutation of the receptor tyrosine kinase gene MERTK in the retinal dystrophic RCS rat. Hum Mol Genet. (2000) 9:645–51. 10.1093/hmg/9.4.645 10699188

[13] Nandrot E Dufour EM Provost AC Pequignot MO Bonnel S Gogat K. Homozygous deletion in the coding sequence of the c-mer gene in RCS rats unravels general mechanisms of physiological cell adhesion and apoptosis. Neurobiol Dis. (2000) 7(6 Pt B):586–99. 10.1006/nbdi.2000.0328 11114258

[14] Vollrath D Feng W Duncan JL Yasumura D D'Cruz PM Chappelow A. Correction of the retinal dystrophy phenotype of the RCS rat by viral gene transfer of Mertk. Proc Natl Acad Sci USA. (2001) 98:12584–9. 10.1073/pnas.221364198 11592982 PMC60097

[15] Feng W Yasumura D Matthes MT LaVail MM Vollrath D. MerTK triggers uptake of photoreceptor outer segments during phagocytosis by cultured retinal pigment epithelial cells. J Biol Chem. (2002) 277:17016–22. 10.1074/jbc.M107876200 11861639

[16] Deng WT Dinculescu A Li Q Boye SL Li J Gorbatyuk MS. Tyrosine-mutant AAV8 delivery of human MERTK provides long-term retinal preservation in RCS rats. Invest Ophthalmol Vis Sci. (2012) 53:1895–904. 10.1167/iovs.11-8831 22408006 PMC3995567

[17] Duncan JL LaVail MM Yasumura D Matthes MT Yang H Trautmann N. An RCS-like retinal dystrophy phenotype in mer knockout mice. Invest Ophthalmol Vis Sci. (2003) 44:826–38. 10.1167/iovs.02-0438 12556419

[18] Young RW. The renewal of photoreceptor cell outer segments. J Cell Biol. (1967) 33:61–72. 10.1083/jcb.33.1.61 6033942 PMC2107286

[19] Young RW Bok D. Participation of the retinal pigment epithelium in the rod outer segment renewal process. J Cell Biol. (1969) 42:392–403. 10.1083/jcb.42.2.392 5792328 PMC2107669

[20] Mullen RJ LaVail MM. Inherited retinal dystrophy: primary defect in pigment epithelium determined with experimental rat chimeras. Science. (1976) 192:799–801. 10.1126/science.1265483 1265483

[21] Edwards RB Szamier RB. Defective phagocytosis of isolated rod outer segments by RCS rat retinal pigment epithelium in culture. Science. (1977) 197:1001–3. 10.1126/science.560718 560718

[22] Tso MO Zhang C Abler AS Chang CJ Wong F Chang GQ. Apoptosis leads to photoreceptor degeneration in inherited retinal dystrophy of RCS rats. Invest Ophthalmol Vis Sci. (1994) 35:2693–9. 8188463

[23] Mazzoni F Muller C DeAssis J Lew D Leevy WM Finnemann SC. Non-invasive *in vivo* fluorescence imaging of apoptotic retinal photoreceptors. Sci Rep. (2019) 9:1590. 10.1038/s41598-018-38363-z 30733587 PMC6367443

[24] Okunuki Y Mukai R Pearsall EA Klokman G Husain D Park DH. Microglia inhibit photoreceptor cell death and regulate immune cell infiltration in response to retinal detachment. Proc Natl Acad Sci USA. (2018) 115:E6264–73. 10.1073/pnas.1719601115 29915052 PMC6142210

[25] Tamm SA Whitcup SM Gery I Wiggert B Nussenblatt RB Kaiser-Kupfer MI. Immune response to retinal antigens in patients with gyrate atrophy and other hereditary retinal dystrophies. Ocul Immunol Inflamm. (2001) 9:75–84. 10.1076/ocii.9.2.75.3972 11449323

[26] Forrester JV. Bowman lecture on the role of inflammation in degenerative disease of the eye. Eye. (2013) 27:340–52. 10.1038/eye.2012.265 23288138 PMC3597872

[27] Whitcup SM Nussenblatt RB Lightman SL Hollander DA Inflammation in retinal disease. Int J Inflam. (2013) 2013:724648 10.1155/2013/724648 24109539 PMC3784265

[28] Peng B Xiao J Wang K So KF Tipoe GL Lin B. Suppression of microglial activation is neuroprotective in a mouse model of human retinitis pigmentosa. J Neurosci. (2014) 34:8139–50. 10.1523/JNEUROSCI.5200-13.2014 24920619 PMC6608244

[29] Gupta N Brown KE Milam AH. Activated microglia in human retinitis pigmentosa, late-onset retinal degeneration, and age-related macular degeneration. Experimental Eye Research. (2003) 76:463–71. 10.1016/S0014-4835(02)00332-9 12634111

[30] Silverman SM Wong WT. Microglia in the retina: roles in development, maturity, and disease. Annu Rev Vis Sci. (2018) 4:45–77. 10.1146/annurev-vision-091517-034425 29852094

[31] Thanos S. Sick photoreceptors attract activated microglia from the ganglion cell layer: a model to study the inflammatory cascades in rats with inherited retinal dystrophy. Brain Res. (1992) 588:21–8. 10.1016/0006-8993(92)91340-K 1393569

[32] Kohno H Koso H Okano K Sundermeier TR Saito S Watanabe S. Expression pattern of Ccr2 and Cx3cr1 in inherited retinal degeneration. J Neuroinflamm. (2015) 12:188. 10.1186/s12974-015-0408-3 26458944 PMC4603985

[33] Kohno H Maeda T Perusek L Pearlman E Maeda A. CCL3 production by microglial cells modulates disease severity in murine models of retinal degeneration. J Immunol. (2014) 192:3816–27. 10.4049/jimmunol.1301738 24639355 PMC4123815

[34] Di Pierdomenico J Garcia-Ayuso D Pinilla I Cuenca N Vidal-Sanz M Agudo-Barriuso M. Early events in retinal degeneration caused by rhodopsin mutation or pigment epithelium malfunction: Differences and similarities. Front Neuroanat. (2017) 11:14. 10.3389/fnana.2017.00014 28321183 PMC5337514

[35] Schlecht A Leimbeck SV Tamm ER Braunger BM. Tamoxifen-containing eye drops successfully trigger cre-mediated recombination in the entire eye. Adv Exp Med Biol. (2016) 854:495–500. 10.1007/978-3-319-17121-0_66 26427451

[36] Sethna S Chamakkala T Gu X Thompson TC Cao G Elliott MH. Regulation of phagolysosomal digestion by caveolin-1 of the retinal pigment epithelium is essential for vision. J Biol Chem. (2016) 291:6494–506. 10.1074/jbc.M115.687004 26814131 PMC4813570

[37] Nagineni CN Kommineni VK Ganjbaksh N Nagineni KK Hooks JJ Detrick B. Inflammatory cytokines induce expression of chemokines by human retinal cells: role in chemokine receptor mediated age-related macular degeneration. Aging Dis. (2015) 6:444–55. 10.14336/AD.2015.0323 26618046 PMC4657816

[38] Duncan DS McLaughlin WM Vasilakes N Echevarria FD Formichella CR Sappington RM. Constitutive and stress-induced expression of CCL5 machinery in rodent retina. J Clin Cell Immunol. (2017) 8:506. 10.4172/2155-9899.1000506 28936366 PMC5604884

[39] Mori I Imai Y Kohsaka S Kimura Y. Upregulated expression of Iba1 molecules in the central nervous system of mice in response to neurovirulent influenza A virus infection. Microbiol Immunol. (2000) 44:729–35. 10.1111/j.1348-0421.2000.tb02556.x 11021405

[40] Li L Eter N Heiduschka P. The microglia in healthy and diseased retina. Exp Eye Res. (2015) 136:116–30. 10.1016/j.exer.2015.04.020 25952657

[41] Langmann T. Microglia activation in retinal degeneration. J Leukoc Biol. (2007) 81:1345–51. 10.1189/jlb.0207114 17405851

[42] Ward HW. Anti-oestrogen therapy for breast cancer: a trial of tamoxifen at two dose levels. Br Med J. (1973) 1:13–4. 10.1136/bmj.1.5844.13 4567104 PMC1588574

[43] Feil S Valtcheva N Feil R. Inducible Cre mice. Methods Mol Biol. (2009) 530:343–63. 10.1007/978-1-59745-471-1_18 19266339

[44] Trinconi CT Reimao JQ Bonano VI Espada CR Miguel DC Yokoyama-Yasunaka JKU. Topical tamoxifen in the therapy of cutaneous leishmaniasis. Parasitology. (2018) 145:490–6. 10.1017/S0031182017000130 28274283

[45] Boneva SK Gross TR Schlecht A Schmitt SI Sippl C Jagle H Cre recombinase expression or topical tamoxifen treatment do not affect retinal structure and function, neuronal vulnerability or glial reactivity in the mouse eye. Neuroscience. (2016) 325:188–201. 10.1016/j.neuroscience.2016.03.050 27026593

[46] Nayfield SG Gorin MB. Tamoxifen-associated eye disease: a review. J Clin Oncol. (1996) 14:1018–26. 10.1200/JCO.1996.14.3.1018 8622006

[47] Wang X Zhao L Zhang Y Ma W Gonzalez SR Fan J. Tamoxifen provides structural and functional rescue in murine models of photoreceptor degeneration. J Neurosci. (2017) 37:3294–310. 10.1523/JNEUROSCI.2717-16.2017 28235894 PMC5373119

[48] van Rooijen N Sanders A van den Berg TK. Apoptosis of macrophages induced by liposome-mediated intracellular delivery of clodronate and propamidine. J Immunol Methods. (1996) 193:93–9. 10.1016/0022-1759(96)00056-7 8690935

[49] van Rooijen N van Kesteren-Hendrikx E. “In vivo” depletion of macrophages by liposome-mediated “suicide”. Methods Enzymol. (2003) 373:3–16. 10.1016/S0076-6879(03)73001-8 14714393

[50] Huang Y Li Z van Rooijen N Wang N Pang CP Cui Q. Different responses of macrophages in retinal ganglion cell survival after acute ocular hypertension in rats with different autoimmune backgrounds. Exp Eye Res. (2007) 85:659–66. 10.1016/j.exer.2007.07.020 17825287

[51] Kataoka K Nishiguchi KM Kaneko H van Rooijen N Kachi S Terasaki H. The roles of vitreal macrophages and circulating leukocytes in retinal neovascularization. Invest Ophthalmol Vis Sci. (2011) 52:1431–8. 10.1167/iovs.10-5798 21051720

[52] Honda M Asai T Oku N Araki Y Tanaka M Ebihara N. Liposomes and nanotechnology in drug development: focus on ocular targets. Int J Nanomed. (2013) 8:495–503. 10.2147/IJN.S30725 23439842 PMC3576887

[53] Faber C Juel HB Jensen BAH Christensen JP Prause JU Thomsen AR. Chemokine expression in murine rpe/choroid in response to systemic viral infection and elevated levels of circulating interferon-gamma. Invest Ophthalmol Vis Sci. (2019) 60:192–201. 10.1167/iovs.18-25721 30654385

[54] Magone MT Whitcup SM. Mechanisms of intraocular inflammation. Chem Immunol. (1999) 73:90–119. 10.1159/000058742 10590576

[55] Yoshida N Ikeda Y Notomi S Ishikawa K Murakami Y Hisatomi T. Laboratory evidence of sustained chronic inflammatory reaction in retinitis pigmentosa. Ophthalmology. (2013) 120:e5–12. 10.1016/j.ophtha.2012.07.006 22986110

[56] McMurtrey JJ Tso MOM. A review of the immunologic findings observed in retinitis pigmentosa. Surv Ophthalmol. (2018) 63:769–81. 10.1016/j.survophthal.2018.03.002 29551596

[57] Blank T Goldmann T Koch M Amann L Schon C Bonin M. Early microglia activation precedes photoreceptor degeneration in a mouse model of CNGB1-linked retinitis pigmentosa. Front Immunol. (2017) 8:1930. 10.3389/fimmu.2017.01930 29354133 PMC5760536

[58] Sasahara M Otani A Oishi A Kojima H Yodoi Y Kameda T. Activation of bone marrow-derived microglia promotes photoreceptor survival in inherited retinal degeneration. Am J Pathol. (2008) 172:1693–703. 10.2353/ajpath.2008.080024 18483210 PMC2408428

[59] Zhao L Zabel MK Wang X Ma W Shah P Fariss RN. Microglial phagocytosis of living photoreceptors contributes to inherited retinal degeneration. EMBO Mol Med. (2015) 7:1179–97. 10.15252/emmm.201505298 26139610 PMC4568951

[60] Scott RS McMahon EJ Pop SM Reap EA Caricchio R Cohen PL. Phagocytosis and clearance of apoptotic cells is mediated by MER. Nature. (2001) 411:207–11. 10.1038/35075603 11346799

[61] Fourgeaud L Traves PG Tufail Y Leal-Bailey H Lew ED Burrola PG. TAM receptors regulate multiple features of microglial physiology. Nature. (2016) 532:240–4. 10.1038/nature17630 27049947 PMC5358512

[62] Nagata S. Apoptosis and clearance of apoptotic cells. Annu Rev Immunol. (2018) 36:489–517. 10.1146/annurev-immunol-042617-053010 29400998

[63] He J Zhao C Dai J Weng CH Bian BSJ Gong Y. Microglia mediate synaptic material clearance at the early stage of rats with retinitis pigmentosa. Front Immunol. (2019) 10:912. 10.3389/fimmu.2019.00912 31105708 PMC6499027

[64] Hafler BP. Clinical progress in inherited *retinal* degenerations: gene therapy clinical trials and advances in genetic sequencing. Retina. (2017) 37:417–23. 10.1097/IAE.0000000000001341 27753762 PMC5465814

[65] Garafalo AV Cideciyan AV Heon E Sheplock R Pearson A WeiYang Yu C. Progress in treating inherited retinal diseases: early subretinal gene therapy clinical trials and candidates for future initiatives. Prog Retin Eye Res. (2019) 2019:100827. 10.1016/j.preteyeres.2019.100827 31899291 PMC8714059

[66] Burstyn-Cohen T. TAM receptor signaling in development. Int J Dev Biol. (2017) 61:215–224. 10.1387/ijdb.160285tb 28621419

