# Supplementary material for: Microglia Inhibition Delays Retinal Degeneration Due to MerTK Phagocytosis Receptor Deficiency
Source: Front Immunol. 2020 Jul 16;11:1463. doi: 10.3389/fimmu.2020.01463 (PMC7381113; doi:10.3389/fimmu.2020.01463)
Supplement: Supplementary file 1 [file Data_Sheet_1.PDF]

Supplementary Material

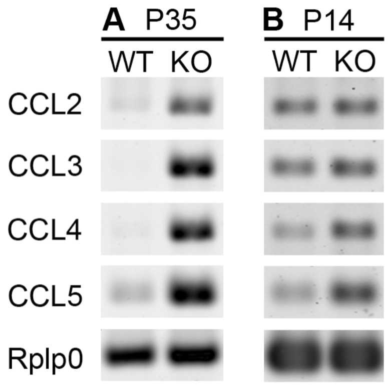

SUPPLEMENTARY FIGURE 1 | CCL4 and CCL5 mRNA overexpression begins before eye opening in *mertk*$^{-/-}$ mouse retina.

Representative RT-PCR products for the cytokines CCL2, CCL3, CCL4 and CCL5 and the housekeeping gene Rplp0 are shown at P35 (**A**), and P14 (**B**) for WT and *mertk*$^{-/-}$ (KO) mouse retina tissues, as indicated. One example experiment is shown from three experiments performed on three biological samples. For each sample, neural retinas from both eyes of two mice were processed. Primers used are listed in Table 1.

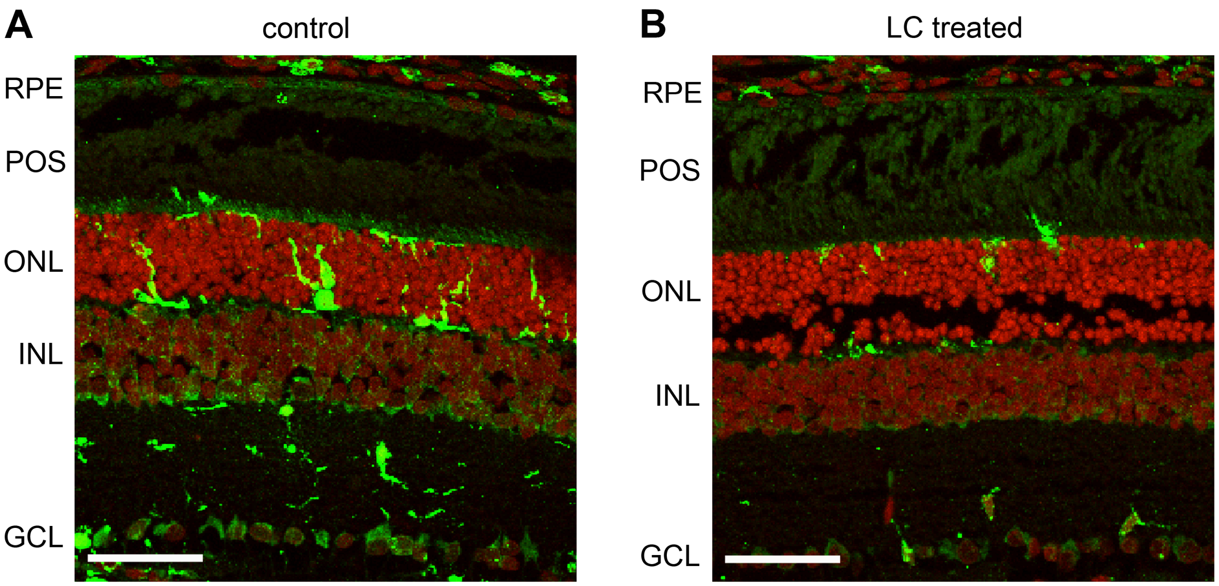

SUPPLEMENTARY FIGURE 2 | A pilot experiment supports efficacy of our LC treatment regimen in reducing retinal microglia migration in the RCS rat.
RCS rats received one intravitreal injection of LC and PBS into contralateral eyes the day after eye opening and weekly i.p. LC injections as described in Materials and Methods before sacrifice at P30 and tissue processing for Iba-1 fluorescence microscopy. Images show Iba-1 (green and cell nuclei counterstain (red) in representative fields of one PBS injected eye (A) and one LC injected eye (B) of 5 littermates tested. In this single experiment, every animal showed reduced outer retinal microglia comparing the LC injected to the PBS injected eye, with extent of reduction ranging from 35% to 56%. Scale bars: 40 µm.

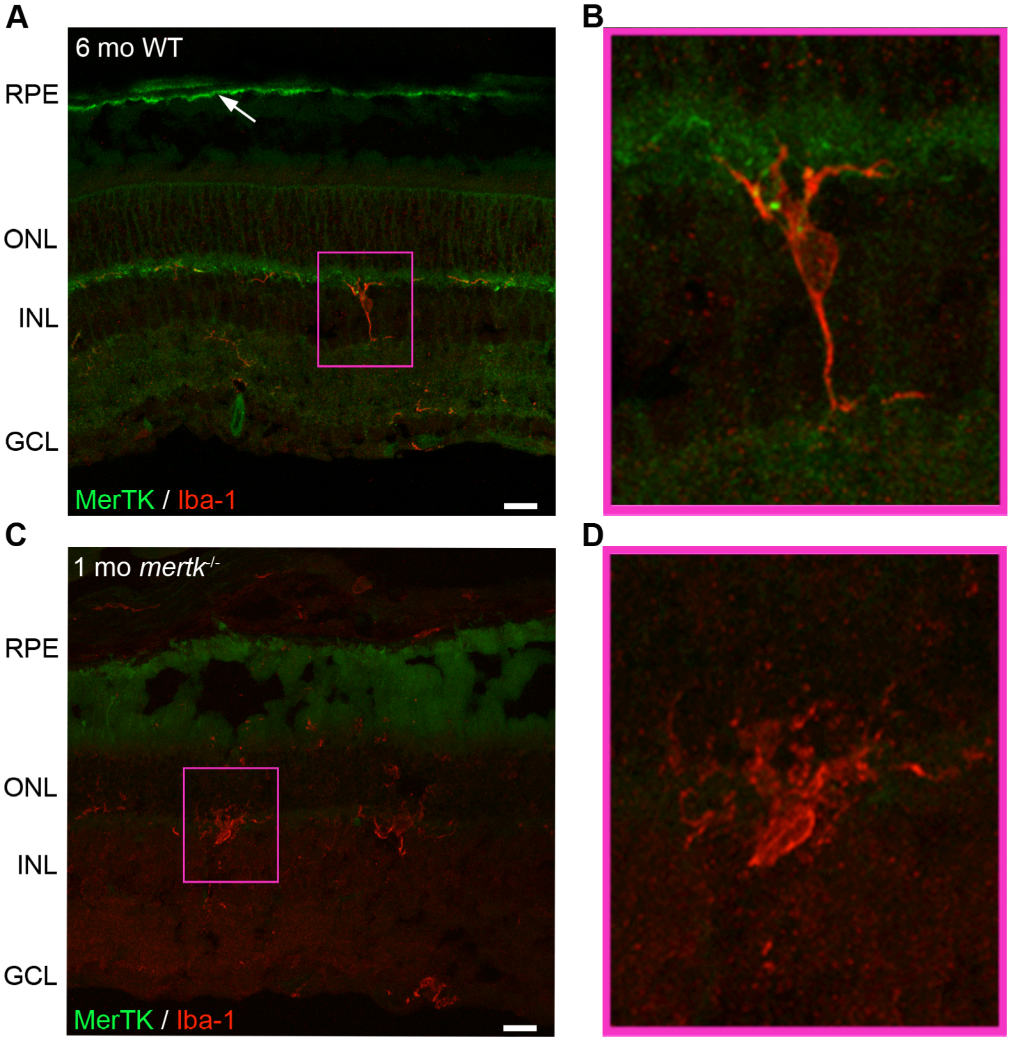

SUPPLEMENTARY FIGURE 3 | Immunofluorescence microscopy does not support abundant MerTK protein in Iba-1-positive microglia in adult WT mouse retina.
12 µm-thick frozen cross sections of whole eyes without lens of 6 month-old (6 mo) WT mice (A,B) or of 1 month-old (1 mo) *mertk*$^{-/-}$ mice (C,D) were stained to label Iba-1-positive microglia (as described in main manuscript, red) and MerTK (rat anti mouse MerTK, Invitrogen #53-5751-80, 1:200, green). Scale bars: 20 µm. **(A)** Representative field showing entire WT retina; arrow indicates MerTK at the apical, phagocytic surface of the RPE. **(B)** Magnification of area as indicated in purple in A. **(C)** Representative field showing entire *mertk*$^{-/-}$ retina as control for antibody specificity. **(D)** Magnification of area as indicated in purple in C.
